# Prediction of Functional Consequences of Missense Mutations in ANO4 Gene

**DOI:** 10.3390/ijms22052732

**Published:** 2021-03-08

**Authors:** Nadine Reichhart, Vladimir M. Milenkovic, Christian H. Wetzel, Olaf Strauß

**Affiliations:** 1 Experimental Ophthalmology, Department of Ophthalmology, Charité—Universitätsmedizin Berlin, Corporate Member of Freie Universität, Berlin Institute of Health, Humboldt-University, 10117 Berlin, Germany; olaf.strauss@charite.de; 2 Molecular Neurosciences, Department of Psychiatry and Psychotherapy, University of Regensburg, 93053 Regensburg, Germany; vladimir.milenkovic@ukr.de (V.M.M.); christian.wetzel@ukr.de (C.H.W.)

**Keywords:** SNP, ANO4 gene, ANO4 protein, disease-association, homology model, protein structure, protein stability, molecular dynamics simulation

## Abstract

The anoctamin (TMEM16) family of transmembrane protein consists of ten members in vertebrates, which act as Ca^2+^-dependent ion channels and/or Ca^2+^-dependent scramblases. ANO4 which is primarily expressed in the CNS and certain endocrine glands, has been associated with various neuronal disorders. Therefore, we focused our study on prioritizing missense mutations that are assumed to alter the structure and stability of ANO4 protein. We employed a wide array of evolution and structure based in silico prediction methods to identify potentially deleterious missense mutations in the ANO4 gene. Identified pathogenic mutations were then mapped to the modeled human ANO4 structure and the effects of missense mutations were studied on the atomic level using molecular dynamics simulations. Our data show that the G80A and A500T mutations significantly alter the stability of the mutant proteins, thus providing new perspective on the role of missense mutations in ANO4 gene. Results obtained in this study may help to identify disease associated mutations which affect ANO4 protein structure and function and might facilitate future functional characterization of ANO4.

## 1. Introduction

The family of anoctamins, known also as TMEM16 proteins, includes 10 members of Ca^2+^-dependent transmembrane proteins [1,2,3,4,5] that exhibit dual function as scramblases and ion channels. Indeed, it is the individual proportion of scramblase as well as ion channel property and activity, which distinguishes each family member. Recent work indicates that the intracellular ion channel activity of anoctamins serve as functional regulators of intracellular Ca^2+^ stores [6,7]. While ANO1 and ANO2 form Ca^2+^-dependent Cl^-^ channels with negligible scramblase activity [2,8,9], ANO6 forms a non-selective monovalent cation channel with predominant scramblase activity [3,4,6,10]. The so far identified ion channel properties are related to either Ca^2+^-activated Cl^−^ channels or Ca^2+^-activated cation channels [11]. The double function of being ion channel and scramblase impedes the clarification of the specific ion channel function [4] because the scramblase itself might activate other non-anoctamin ion channel currents superimposing the currents through the anoctamin pore. Among the family members, ANO1, ANO2, ANO5 and ANO6 are best characterized, because they are associated with various diseases. For example, mutations in ANO6 lead to Scott’s syndrome [3,4], ANO1 expression relates to cancer, is involved in cystic fibrosis and colon disease [12,13,14], and loss of ANO2 function impairs olfactory perception [15,16]. Mutations in ANO5 cause a variety of muscle dystrophies [17].

Among the anoctamin family, ANO4 is not well studied. Using a side-directed mutagenesis approach, we demonstrated that ANO4 forms a Ca^2+^-dependent non-selective monovalent cation channel [11]. Although most of the reports show that ANO4 is found in the plasma membrane it exhibited intracellular function with relevance for Ca^2+^ store filling [6]. Additionally, recently, literature reported a link between ANO4 expression and some disease mechanisms. The group of Brown and colleagues suggested that ANO4 might be involved in the regulation of aldosterone secretion [18,19]. Furthermore, active myelin-lesions in multiple sclerosis display increased levels of ANO4 expression [20]. Support for a significant role of ANO4 in disease rises from genetic analysis. Polymorphism detection and single nucleotide polymorphism-directed (SNP) genome-wide analysis revealed that ANO4 provides a genetic background for a variety of brain disorders, such as schizophrenia, Alzheimer’s disease and anxiety disorders [21,22,23,24]. A meta-analysis of genome-wide association studies in breast cancer revealed an association with a cluster of genes that is involved in endo-/exocytosis, among them ANO4 [25]. The recent work revealed a more specific function of ANO4 as being a Ca^2+^-dependent cation channel with involvement in Ca^2+^ signaling, in plasma membranes and membranes of organelles [11]. In addition, recently published data connect ANO4 to numerous disease scenarios. Thus, using an in silico approach, we aimed to systematically investigate most frequently occurring genetic variants in the ANO4 gene by several well-established computational methods. Mutations, which were predicted to be deleterious by seven different computational tools, were further analyzed using homology modeling. To examine the effect of missense mutations on atomic level, deleterious mutations were mapped to the modeled human ANO4 structure and subjected to 60 ns molecular dynamics simulations (MDS). Our data show that both G80A and A500T mutations significantly alter the flexibility and the stability of the mutant protein.

To the best of our knowledge, this study is the first extensive in silico analysis that combines polymorphism analysis and a molecular dynamics approach for predicting clinical relevance of ANO4 nsSNPs.

## 2. Results

### 2.1. SNP Dataset Collection

**Figure 1:**
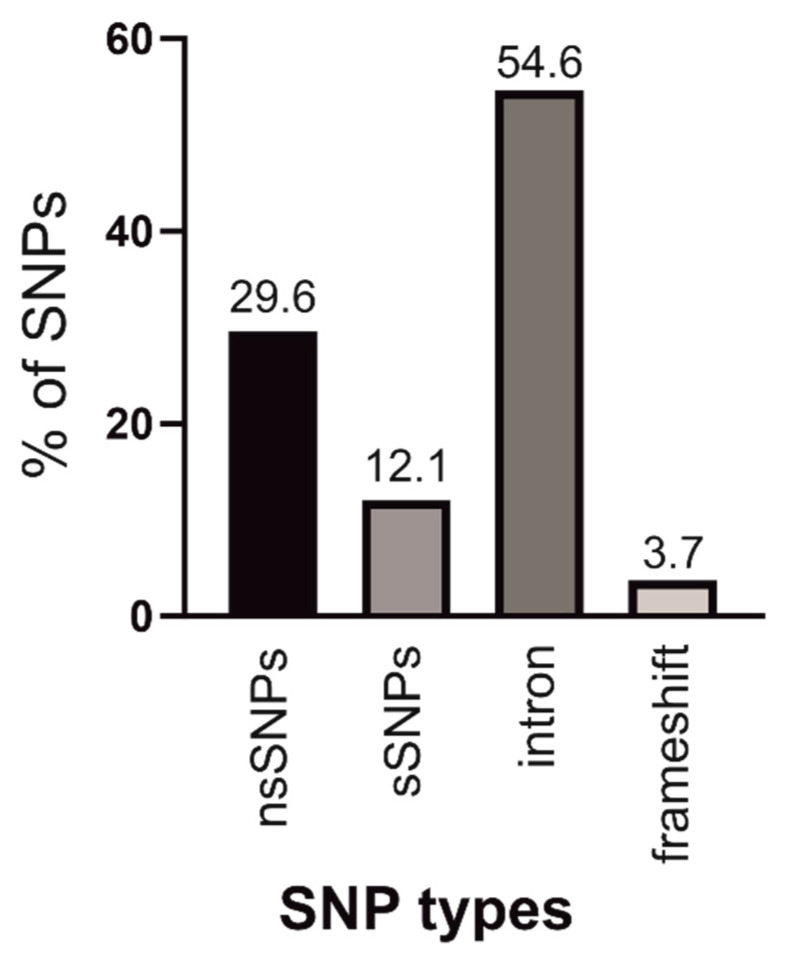
Distribution of coding nsSNPs, coding sSNPs, intronic SNPs, and SNPs causing frameshift in the human ANO4 gene.

The human ANO4 gene reveals a total of 1615 genetic variants in a data set from dbSNP and gnomAD database (Figure 1 and Table S1). The majority of the SNPs are located in intronic regions (881 variants; 54.6%). The ANO4 gene contains 194 (12.1%) synonymous SNPs and 478 (29.6%) non-synonymous SNPs (nsSNPs), which are evenly distributed across the protein coding sequence. ANO4 transcripts are predominantly expressed in the CNS, certain genital organs (cervix, ovaries and prostate) and in adrenal glands (Figure S1). Thus, SNPs in ANO4 might result in pathologies/diseases of the CNS or affect the function of genital organs and the secretion by the adrenal gland. Since most of the nsSNPs in the ANO4 gene have a very low global minor allele frequency in human populations (Table S1), we have selected 15 most frequent nsSNPs for subsequent analysis.

### 2.2. Detection of Potentially Deleterious Mutations on ANO4 Structure and Function

**Figure 2:**
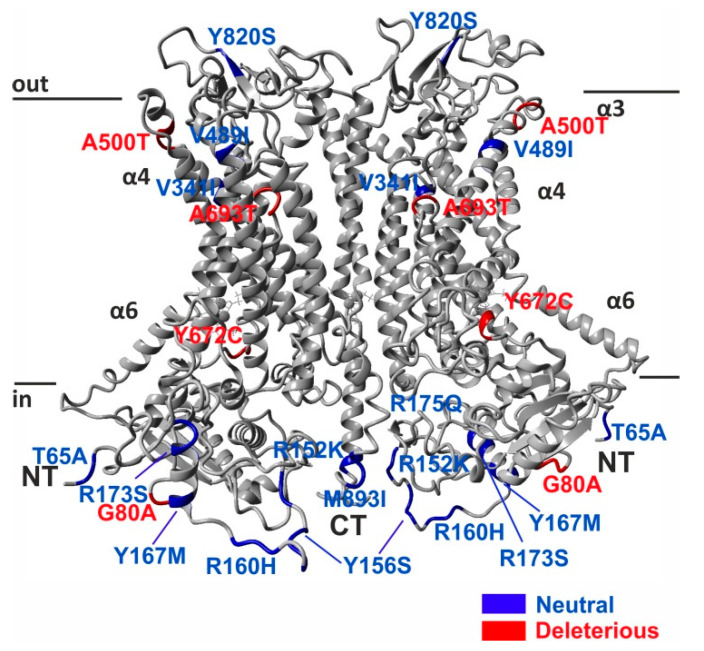
Identification of deleterious nsSNPs in human ANO4 gene. Non-deleterious (blue) and deleterious (red) nsSNPs were mapped to the structure of human ANO4 protein structure obtained from homology modeling.

To further substantiate which nsSNPs might have impact on ANO4 protein structure and stability, and to increase the confidence in prediction of deleterious mutations, we have incorporated 7 most commonly used algorithms (Provean, Polyphen-2, SNAP2, Mutpred 2.0, SNPs&GO, PhD-SNP, SIFT) in this study. Of the 15 most frequent nsSNPs in the ANO4 gene, four ANO4 variants were predicted to consistently affect the stability of ANO4 protein by the majority of algorithms used: G80A, A500T, Y672C, and A693T (Table 1 and Figure 2). According to the evolutionary conservation prediction tool ConSurf, all 4 potentially deleterious mutations are in evolutionary highly conserved regions of the ANO4 protein, suggesting their functional importance (Figure S2).

### 2.3. Homology Model of *ANO4*

In general, amino acid substitutions can alter protein structure, folding or stability, all of which are critical for protein function. Therefore, knowledge of a ANO4 3D structure is indispensable for better understanding the functionality of the mutated protein. Since the crystal structure of human ANO4 has not been revealed so far, we used a homology approach to model human wild-type (WT) and mutant ANO4 structures. The crystal structure of Mus musculus ANO4 (6QP6), which was solved at 3.2 Å resolution shows a protein sequence identity of 53.6%, and the sequence similarity of 72.6% served as a template for homology modeling. The 16 loop regions which could not be aligned to mouse ANO4 were additionally modelled.

Subsequently, the ANO4 structure was subjected to a combined steepest descent and simulated annealing minimization, and the quality of the model structure was evaluated by considering the overall Z-score. G80A, A500T, Y672C, and A693T mutations were mapped to the native ANO4 structure using Yasara software. The initial WT and mutant ANO4 structures were further refined and subjected to an energy minimization algorithm.

### 2.4. Molecular Dynamics Simulation Analysis of Native and Mutant *ANO4* Protein

**Figure 3:**
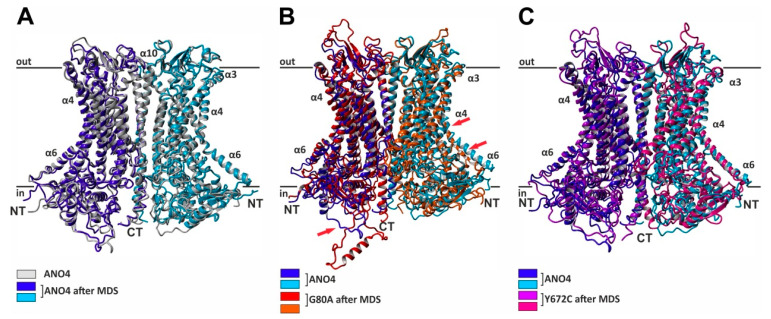
Alignment of the native and mutant ANO4 structures. Ribbon representation of a superposition of ANO4 structures before and after 60 ns of MDS. Subunits are shown in dark and light shades of the corresponding color. Native ANO4 structure before MDS was aligned with (**A**) ANO4 WT, (**B**) G80A, and (**C**) Y672C structures, respectively, after 60 ns of MDS. The mutation at position G80A, which is located in the N-terminal region of the protein destabilized the whole protein, especially the N terminus and the α-helices 4 and 6 (red arrows). The Y672C mutation does not affect the stability of ANO4 protein.

**Figure 4:**
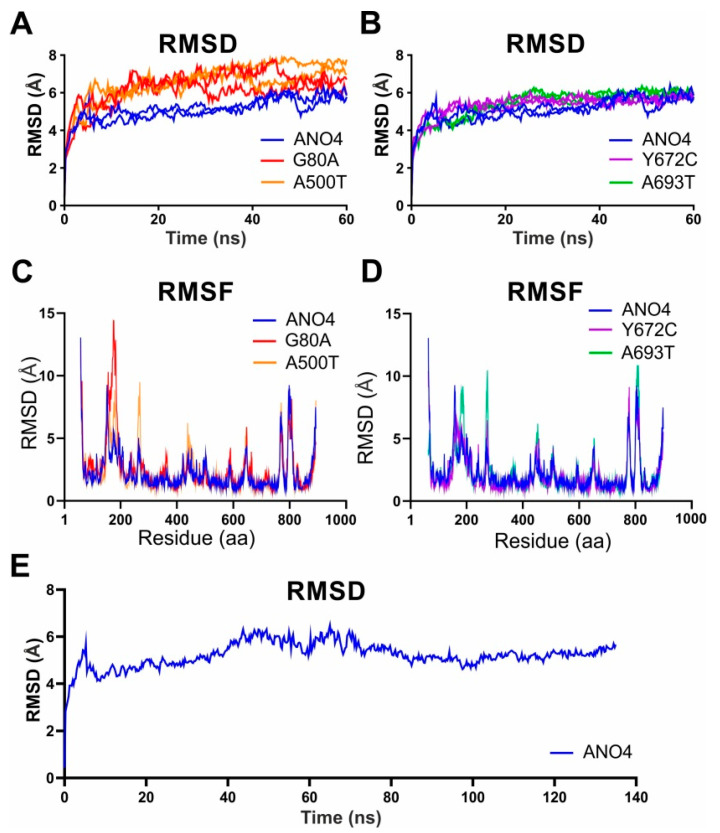
Molecular dynamics simulation of native and mutant ANO4 structures. (**A**,**B**) The RMSD values of native and mutant structures from two independent simulations are shown as a function of time after 60 ns of MDS. G80A and A500T structures exhibited significantly increased structural deviation in comparison to ANO4 WT, Y672C and A693T mutant structures. (**C**,**D**) RMSF of the central alpha carbon per residue over entire simulation of native and mutant structures. (**E**) The stability of native ANO4 structure was evaluated during 135 ns of MDS, showing that ANO4 WT structure reached equilibrium after 10 ns and remained stable afterwards.

**Figure 5:**
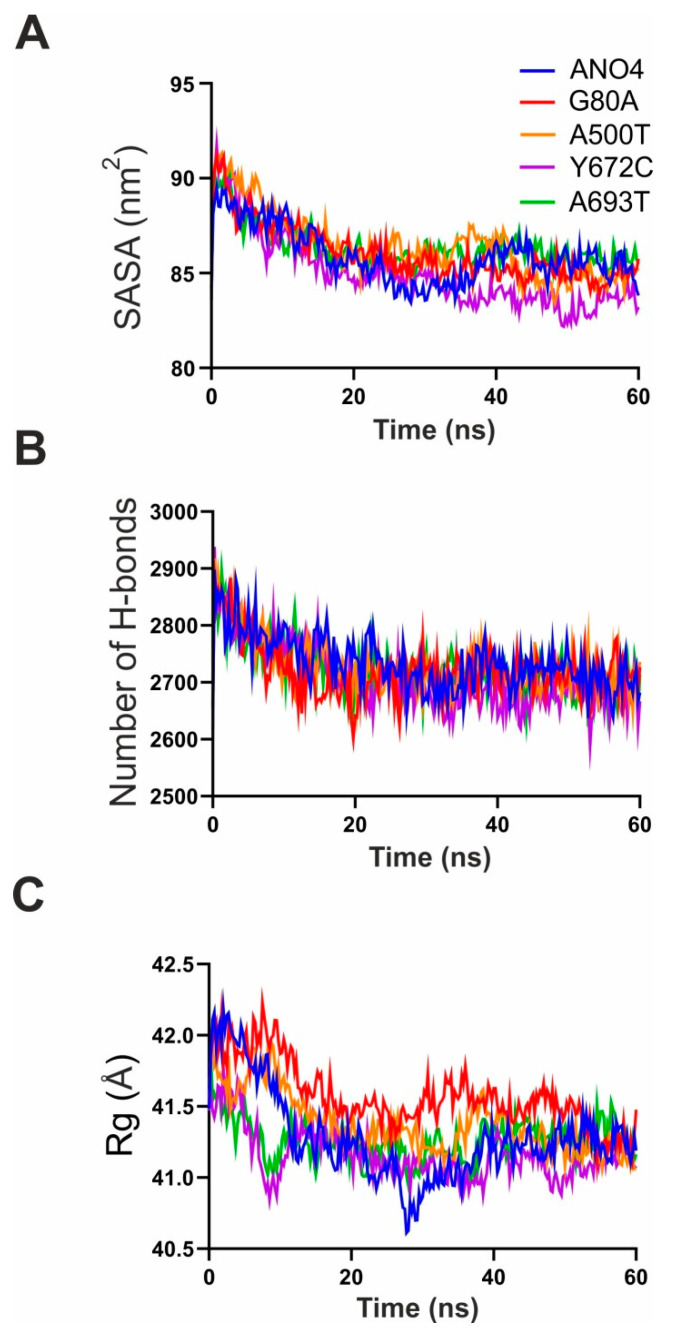
Stability of the ANO4 WT and mutant structures during MDS was assessed by means of (**A**) solvent accessible surface area, SASA, (**B**) number of H-bonds, and (**C**), radius of gyration, Rg.

In order to obtain a more detailed insight into the effect of missense mutations on ANO4 structure and stability, we implemented a molecular dynamics simulation (MDS), which has been used to reveal the impact of mutations on the stability of protein structure at atomic level, using refined native and mutant protein structures. Two independent simulations were carried out for each native and mutant structure, respectively. To compare possible structural consequences of missense mutations, we have performed spatial superposition of the native and mutant structures before and after the molecular dynamics simulation (Figure 3). Native ANO4 protein aligned very well before and after MDS (Figure 3A), similarly as Y672C (Figure 3B) and A693T (data not shown). On the other hand mutation G80A destabilized the whole protein especially N-terminus and α-helices 4 and 6 (Figure 3C). According to the RMSD plot (Figure 4A,B and Table 2), WT and both Y672C and A693T mutant structures reached equilibrium state after 10 ns, whereas RMSD values of G80A, and A500T mutant were notably higher (Figure 4A and Table 2). The trajectory files generated from the last 50 ns run of MDS were subsequently analyzed using the Yasara software package. Root mean square deviation (RMSD), root mean square fluctuation (RMSF), solvent-accessible surface area (SASA), number of H-bonds, and radius of gyration (Rg) were assessed in ANO4 WT and the four missense mutations. First, changes in RMSD, indicative of overall protein stability, were measured. WT, Y672C and A693T showed similar values (5.02 ± 0.57Å, 5.36 ± 0.59Å, 5.39 ± 0.77Å), whereas the RMSD values for G80A (6.19 ± 0.88Å) and A500T (6.54 ± 0.97Å) were significantly higher than for the native ANO4 (Figure 3A,C, and Table 2). The increase in RMSD and thus the increase in structural deviation is indicative of the reduction in the stability of the protein. The values of RMSF (Figure 4C,D), that reflects the structural flexibility of the protein, did not differ among WT and the four mutants. The same applied for Rg, SASA, and number of H-bonds (Figure 5 and Table 2) suggesting similar protein dimensions of the mutant structures compared to ANO4 WT. In conclusion, our MDS analysis suggests that both G80A and A500T mutations have higher structural flexibility when compared to the native protein, causing significant structural changes in the mutant protein. On the other hand, Y672C and A693T mutations, which were predicted to be deleterious as well, had no effect on the stability of mutant proteins.

## 3. Discussion

ANO4 shows distinct expression patterns in the human body, implying that changes in its expression activity or in ANO4 gene nucleotide sequence would result in specific organotypic diseases. Thus, we implemented a systematic in silico analysis to identify ANO4 gene polymorphisms, as well as vulnerable sites that affect ANO4 structure. As a result, we identified structural parameters affected by deleterious SNPs with potential significant impact to ANO4 protein stability, whereas parameters indicative for structural flexibility and radius of gyration were not affected. By comparison of the SNP location of mutations with no effect to those with strong impact for protein stability, we identified homology regions in ANO4 that represent sites with highest potential of disease association. These regions might help to judge the potential severity of disease-associated SNPs.

The selection of known SNPs in the ANO4 gene from the gnomAD database has already revealed a large number of known SNPs. Furthermore, we obtained the pattern of preferential ANO4 expression in the different organs (Figure S1). Already at the level of nucleotide alterations, SNPs with a higher disease potential were easily identified. More than 400 non-synonymous SNPs were identified in coding regions of the ANO4 gene were identified as promising candidates. As this number makes almost 30% of all known SNPs, the ANO4 gene bears a strong potential to reveal disease associations. Compared to other members of the anoctamin family, such as ANO1, ANO2 and ANO6, the function and pathophysiology of ANO4 is rather poorly investigated. At the level of nucleotide analysis of known SNPs in ANO4, a role of this ion channel in disease is substantiated.

However, we found that more than 400 non-synonymous SNPs were evenly distributed over the nucleotide sequence of ANO4. This makes it difficult to judge their potential impact on ANO4 function and for a true disease-association. To address this question, we performed an in silico approach to evaluate the impact of non-synonymous SNPs. Based on known ANO structures and identification of homology regions with high evolutionary stability, we first generated a homology model of the three-dimensional structure of the human ANO4 protein. This model was tested for the impact of SNPs on protein structure by seven different algorithms that resulted in four variants with a predicted high deleterious effect on protein structure. These SNPs localized to evolutionary highly conserved homology areas, thereby predicting the severity of functional impairment of ANO4, and its related disease potential. To give an estimate of the effect of the mutation (SNP) onto the protein structure, we performed MD simulations to determine structural significance of these SNPs with potential function. MD simulation became a broadly used, valuable tool to distinguish the disease-causing SNPs from the neutral SNPs [26,27,28,29]. Various parameters that predict protein stability were calculated in the last 60 ns of the simulation trajectories: RMSD, RMSF, radius of gyration, solvent-accessible surface area and number of H-bonds. Only the parameter, which predicts structural deviation, was altered in the two variants. G80A and A500T showed larger deviation than the ANO4 WT, which became obvious by comparing the homology models of native ANO4 and G80A with the variant Y672C which shows a mean of deviation comparable to WT: the structure of G80A differs strongly from that of WT or Y672C ANO4. In further studies MD simulations of a null mutation G80V might help to further substantiate the implications of amino acid position 80 for protein stability of ANO4, given the similar hydrophobic property of alanine and valine.

We used MD simulations of 60 ns that were used by other authors for MD simulations too. In a similar approach using MDS for 50 ns, Agrahari et al. found differences either in RMSD, RMSF and radius of gyration when comparing several mutations of a protein involved in Lesch–Nyhan disease. The same applied for GJB1, that is associated with X-linked Charcot-Marie-tooth diesease [27,28]. With ANO4 WT we performed a prolonged MD simulation of 135 ns that shows that the structural parameters have already stabilized after a little more than 10 ns. Thus, we are in good agreement of the usefulness of shorter MD simulation durations.

Using homology modeling as a starting point for structural studies has its own limitations, however high protein sequence identity with homologs from different species with known crystal structure (in this case 53.6% identity of murine ANO4 to the human ANO4), usually warranties reliable predictions [30,31]. Thus, the approach to use a homology model for prediction of mutant protein stability by computer-based analysis is common when the crystal structure of the protein of interest has not been reported so far [26,28,29].

In summary, we found that SNPs in evolutionary highly conserved homology regions of ANO4 alter the protein stability and might lead to a loss or reduction of ANO4 activity in tissues with high ANO4 expression, such as genital organs (cervix, ovaries, prostate), tissues with secretory activity (adrenal gland) and the central nervous system. Indeed, SNPs in the ANO4 gene associate with breast cancer and various brain disorders such as schizophrenia or anxiety disorders. Based on our investigation, we suggest that these associations result mainly from reduced or loss of ANO4 activity in the affected tissues. The recently reported role of ANO4 in the regulation of aldosterone secretion fits into the observation that ANO4 is preferentially expressed in tissue with secretory activity [18,19].

In the present study, we were able to identify regions in the ANO4 protein, which we predicted to be highly impacted by SNPs. This information might be useful for identifying disease-associated SNPs.

## 4. Materials and Methods

### 4.1. Dataset Collection

SNPs of the human ANO4 gene were retrieved from the National Center for Biotechnology Information (NCBI) SNPs database, (dbSNP) [32] and genome aggregation database (gnomAD) database [33], which contains data from 125,748 exomes and 15,708 whole genomes. All redundant, outdated and SNPs found in non-canonical transcripts were removed from further analysis. The amino acid sequence of human ANO4 (accession number: NP_849148.2) was retrieved from the NCBI website.

The Genotype-Tissue Expression (GTEx) project was supported by the Common Fund of the Office of the Director of the National Institutes of Health, and by NCI, NHGRI, NHLBI, NIDA, NIMH, and NINDS. The data used for the analyses described in this manuscript were obtained from the GTEx Portal on 15 June 2020.

### 4.2. Computational Methods for Detection of Deleterious Mutations

Seven computational algorithms for prediction of the effect of nsSNPs on the protein structure and stability were used. Protein Variation Effect Analyzer, (PROVEAN) [34], estimates the functional impact of protein sequence variations as “neutral” or “deleterious”. Polymorphism Phenotyping v2 (PolyPhen-2) [35] predicts possible impact of an amino acid substitution on the structure and function of a human protein using straightforward physical and evolutionary comparative considerations. Mutations with probabilistic score bellow 0.15 are classified as benign, the ones in the range of 0.15 to 0.84 are classified as “possibly damaging”, and mutation is classified as “most likely damaging” if the score is higher than 0.85. The sorting intolerant from tolerant (SIFT) method [36], which is based on the degree of conservation of amino acids in sequence alignments, predicts whether an amino acid substitution affects protein function. SIFT assigns scores where a variant with a score less than 0.05 is considered deleterious, whereas a variant with a score greater than 0.05 is considered tolerated. Predictor of human Deleterious Single Nucleotide Polymorphisms (PhD-SNP) [37] is a Support Vector Machine (SVM) single sequence based method which predicts whether an nSNP is a neutral polymorphism or a disease associated polymorphism. Screening for Non-Acceptable Polymorphisms 2 (SNAP2) [38] method utilizes sequence information, secondary structure and residue conservation as well as neural networks to predict whether the mutation is neutral or non-neutral. SNPs&GO [39] is SVM based method which predicts if a given single point protein variation can be classified as disease associated or neutral using evolutionary information, and functions encoded in gene ontology terms. Mutations with a score greater than 0.5 are classified as disease related. We additionally estimated the evolutionary conservation rate of each residue in ANO4 using ConSurf [40], which quantifies the degree of conservation for each amino acid position in a given alignment. The extent of conservation of each residue is presented as a score in the range of 1–9, with 1 denotes highly variable sites, and 9 evolutionary conserved sites.

### 4.3. Homology Modeling of the Human ANO4 Structures

Homology model of human ANO4 was built using YASARA molecular modelling program version 19.9.17 [41]. The hm_build.mcr macro of the YASARA software was used with the default parameters except the maximum oligomerization state was set to two. The amino acid sequence of human ANO4 (accession number: NP_849148.2) was submitted to modeling, and template structures were searched by running six iterations of PSI-BLAST. For the top scoring templates (PDB ID: 6QP6 and 5OYB) from the protein data bank a total of 12 models were created, and the quality of the model structure was evaluated by considering the overall Z-score. The Z-score has been defined as the weighted averages of the individual Z-scores using the formula, Z-score = 0.346 × Dihedrals + −0.809 × Packing1D + 0.4656 × Packing3D [41]. ANO4 model structure with the Z-score of -0.862 which was based on Mus musculus Ano6 structure (PDB ID: 6QP6 solved at 3.2 Å) was subjected to further refinement using the md_refine.mcr macro of YASARA, while the models with lower quality Z-scores were discarded. G80A, A500T, Y672C and A693T mutant structures were obtained by mutating corresponding positions in the ANO4 model structure. After the mutation, the structures were subjected to an energy minimization with the YAMBER force field as described previously [42]. The structures of WT and mutant ANO4 proteins were superimposed and aligned using MUSTANG program implemented in the YASARA software package [43].

### 4.4. Molecular Dynamics Simulation

Molecular dynamics (MD) simulation technique was used in order to understand the effect of point mutations on ANO4 protein structure and stability. MD simulations were run on an AMD R9 3900X @4.20 GHz workstation with 12 computing cores, and 24 threads, with hyper-threading enabled, equipped with 16 GB of RAM. This workstation had one RTX 2080Ti graphic card onboard. GPU card was set up with default values of memory and core clock speeds and voltages. The energy minimized structures of the native and mutant ANO4 proteins were used as starting points for the MD simulations, which were carried out with YASARA software using the md_runmembrane.mcr macro and the AMBER14 force field.

The ANO4 simulation was set up automatically by first scanning the protein for exposed transmembrane helices. Based on the identified secondary structure elements, the protein was oriented normally with respect to the plane of the membrane and the XZ-plane. The equilibrated membrane, consisting of phosphatidylethanolamine molecules was enclosed in the simulation cell of size [69 × 98 × 65] Å and the native or mutant proteins were then embedded into the membrane. The simulation cell contained around 324,217 atoms which included the ANO4 dimer together with phosphatidylethanolamine and water molecules. During the equilibration phase which lasted 250 ps, the membrane was artificially stabilized to avoid distortions while the simulation cell adapted to the pressure exerted by the membrane. The MD was run twice for each system for 60 ns at constant temperature of 298K, and pressure with a time step of 2 fs and snapshots were saved at every 25 ps, as defined in md_runmembrane.mcr macro. The computing time for the simulation was 440 h.

The trajectory files generated by MD simulations were analyzed using YASARA to obtain the root-mean-square deviation (RMSD), root-mean-square-fluctuation (RMSF), solvent-accessible surface area (SASA), number of H-bonds and radius of gyration (Rg) values, which were statistically analyzed to determine the differences in the RMSD, RMSF, and Rg values between native and mutant protein structures.

## Figures and Tables

**Table 1:** In silico analysis of ANO4 nsSNPs used in this study and their predicted Provean, Polyphen-2, SNAP2, Mutpred2.0, SNPs&GO, PhD-SNP, and SIFT scores (B = benign, D = deleterious). Predicted deleterious SNPS are depicted in bold.

SNP ID	Position	Provean	Polyphen-2	SNAP2	Mutpred2.0	SNPs&GO	PhD-SNP	SIFT	Allele Frequency	Consensus 1–4 = B, >5 = D
**rs34162417**	**G80A**	**Deleterious**	**probably damaging**	**neutral**	**Deleterious**	**Disease**	**Disease**	**Deleterious**	**0.047557**	**D (6/7)**
rs116925463	V341I	Neutral	benign	neutral	benign	neutral	neutral	Tolerated	0.002853	B (0/7)
rs201084190	Y156S	Neutral	benign	neutral	Deleterious	Disease	neutral	Tolerated	0.000042	B (2/7)
**rs143089752**	**Y672C**	**Neutral**	**probably damaging**	**effect**	**Deleterious**	**Disease**	**Disease**	**Deleterious**	**0.000366**	**D (6/7)**
rs202022090	Y820S	Neutral	probably damaging	effect	Deleterious	neutral	neutral	Tolerated	0.000283	B (3/7)
rs878855567	R152K	Neutral	benign	neutral	Deleterious	neutral	neutral	Tolerated	0.000278	B (1/7)
**rs150353677**	**A500T**	**Deleterious**	**probably damaging**	**neutral**	**Deleterious**	**Disease**	**Disease**	**Tolerated**	**0.000175**	**D (5/7)**
rs566155639	I167M	Neutral	benign	neutral	benign	neutral	neutral	Tolerated	0.00006	B (0/7)
rs150616124	R173S	Neutral	benign	neutral	benign	neutral	neutral	Tolerated	0.000072	B (0/7)
**rs200450110**	**A693T**	**Deleterious**	**probably damaging**	**effect**	**Deleterious**	**Disease**	**neutral**	**Deleterious**	**0.000159**	**D (5/7)**
rs199651099	R175Q	Neutral	benign	neutral	benign	neutral	neutral	Tolerated	0.000151	B (0/7)
rs201623880	M893I	Neutral	possibly damaging	neutral	Deleterious	neutral	neutral	Tolerated	0.000139	B (1/7)
rs140325859	V489I	Neutral	probably damaging	neutral	Deleterious	neutral	neutral	Tolerated	0.000104	B (2/7)
rs372743717	T65A	Neutral	benign	neutral	benign	neutral	neutral	Tolerated	0.000052	B (0/7)
rs778988665	R160H	Neutral	probably damaging	neutral	Deleterious	neutral	neutral	Tolerated	0.000060	B (2/7)

**Table 2:** Average structural properties calculated for the full length ANO4 wild-type, G80A, A500T, Y672C, and A693T structures and corresponding standard deviations (in parentheses). * Significant at *p* < 0.05 are depicted in bold and with asterisk.

	ANO4 wt	G80A	A500T	Y672C	A693T
Cα-RMSD (Å)	5.02 (0.57)	**6.19** * (0.88)	**6.54** * (0.97)	5.36 (0.59)	5.39 (0.77)
Cα-RMSF (Å)	2.21 (1.62)	2.33 (1.87)	2.27 (1.84)	2.03 (1.44)	2.11 (1.65)
Rg-protein (Å)	41.17 (0.37)	41.64 (0.24)	41.34 (0.26)	41.10 (0.25)	41.10 (0.26)
H-bonds	2700 (57)	2699 (50)	2706 (59)	2669 (62)	2714 (51)
SASA (nm^2^)	85.40 (1.53)	86.31 (1.39)	86.18 (1.77)	85.51 (1.63)	86.02 (1.38)

## Data Availability

ANO4 models created in this study are available on request.

## Supplementary Materials

The following are available online at https://www.mdpi.com/1422-0067/22/5/2732/s1, Figure S1: mRNA tissue expression of ANO4, Table S1: List of ANO4 SNPs used in this study.

## References

[1] Boccaccio A., Di Zanni E., Gradogna A., Scholz-Starke J. (2019). Lifting the veils on TMEM16E function. Channels.

[2] Falzone M.E., Malvezzi M., Lee B.-C., Accardi A. (2018). Known structures and unknown mechanisms of TMEM16 scramblases and channels. J. Gen. Physiol..

[3] Oh U., Jung J. (2016). Cellular functions of TMEM16/anoctamin. Eur. J. Physiol..

[4] Picollo A., Malvezzi M., Accardi A. (2015). TMEM16 Proteins: Unknown Structure and Confusing Functions. J. Mol. Biol..

[5] Milenkovic V.M., Brockmann M., Stöhr H., Weber B.H., Strauss O. (2010). Evolution and functional divergence of the anoctamin family of membrane proteins. BMC Evol. Biol..

[6] Cabrita I., Benedetto R., Fonseca A., Wanitchakool P., Sirianant L., Skryabin B.V., Schenk L.K., Pavenstädt H., Schreiber R., Kunzelmann K. (2017). Differential effects of anoctamins on intracellular calcium signals. FASEB J..

[7] Kunzelmann K., Cabrita I., Wanitchakool P., Ousingsawat J., Sirianant L., Benedetto R., Schreiber R. (2015). Modulating Ca2+ signals: A common theme for TMEM16, Ist2, and TMC. Pflügers Arch..

[8] Tian Y., Schreiber R., Kunzelmann K. (2012). Anoctamins are a family of Ca2+-activated Cl- channels. J. Cell Sci..

[9] Hartzell H.C., Yu K., Xiao Q., Chien L.-T., Qu Z. (2009). Anoctamin/TMEM16 family members are Ca2+-activated Cl−channels. J. Physiol..

[10] Kunzelmann K., Nilius B., Owsianik G., Schreiber R., Ousingsawat J., Sirianant L., Wanitchakool P., Bevers E.M., Heemskerk J.W.M. (2013). Molecular functions of anoctamin 6 (TMEM16F): A chloride channel, cation channel, or phospholipid scramblase?. Pflügers Arch..

[11] Reichhart N., Schöberl S., Keckeis S., AlFaar A.S., Roubeix C., Cordes M., Crespo-Garcia S., Haeckel A., Kociok N., Föckler R. (2019). Anoctamin-4 is a bona fide Ca2+-dependent non-selective cation channel. Sci. Rep..

[12] Ji Q., Guo S., Wang X., Pang C., Zhan Y., Chen Y., An H. (2019). Recent advances in TMEM16A: Structure, function, and disease. J. Cell. Physiol..

[13] Kunzelmann K., Ousingsawat J., Cabrita I., Doušová T., Bähr A., Janda M., Schreiber R., Benedetto R. (2019). TMEM16A in Cystic Fibrosis: Activating or Inhibiting?. Front. Pharmacol..

[14] Crottès D., Jan L.Y. (2019). The multifaceted role of TMEM16A in cancer. Cell Calcium.

[15] Dibattista M., Pifferi S., Boccaccio A.E., Menini A., Reisert J. (2017). The long tale of the calcium activated Cl− channels in olfactory transduction. Channels.

[16] Pifferi S., Cenedese V., Menini A. (2011). Anoctamin 2/TMEM16B: A calcium-activated chloride channel in olfactory transduction. Exp. Physiol..

[17] Silva A.M.S., Coimbra-Neto A.R., Souza P.V.S., Winckler P.B., Gonçalves M.V.M., Cavalcanti E.B.U., Carvalho A.A.D.S., Sobreira C.F.D.R., Camelo C.G., Mendonça R.D.H. (2019). Clinical and molecular findings in a cohort of ANO5 -related myopathy. Ann. Clin. Transl. Neurol..

[18] Maniero C., Scudieri P., Shaikh L.H., Zhao W., Gurnell M., Galietta L.J., Brown M.J. (2019). ANO4 (Anoctamin 4) Is a Novel Marker of Zona Glomerulosa That Regulates Stimulated Aldosterone Secretion. Hypertension.

[19] Maniero C., Zhou J., Shaikh L.H., Azizan E.A.B., McFarlane I., Neogi S., Scudieri P., Galietta L.J., Brown M.J. (2015). Role of ANO4 in regulation of aldosterone secretion in the zona glomerulosa of the human adrenal gland. Lancet.

[20] Hendrickx D.A.E., Van Scheppingen J., Van Der Poel M., Bossers K., Schuurman K.G., Van Eden C.G., Hol E.M., Hamann J., Huitinga I. (2017). Gene Expression Profiling of Multiple Sclerosis Pathology Identifies Early Patterns of Demyelination Surrounding Chronic Active Lesions. Front. Immunol..

[21] Sherva R., Tripodis Y., Bennett D.A., Chibnik L.B., Crane P.K., de Jager P.L., Farrer L.A., Saykin A.J., Shulman J.M., Naj A. (2014). Genome-wide association study of the rate of cognitive decline in Alzheimer’s disease. Alzheimers Dem..

[22] Webb B.T., Guo A.-Y., Maher B.S., Zhao Z., Oord E.J.V.D., Kendler K.S., Riley B.P., Gillespie N.A., Prescott C.A., Middeldorp C.M. (2012). Meta-analyses of genome-wide linkage scans of anxiety-related phenotypes. Eur. J. Hum. Genet..

[23] Terracciano A., Sanna S., Uda M., Deiana B., Usala G., Busonero F., Maschio A., Scally M., Patriciu N., Chen W.-M. (2008). Genome-wide association scan for five major dimensions of personality. Mol. Psychiatry.

[24] Athanasiu L., Mattingsdal M., Kähler A.K., Brown A., Gustafsson O., Agartz I., Giegling I., Muglia P., Cichon S., Rietschel M. (2010). Gene variants associated with schizophrenia in a Norwegian genome-wide study are replicated in a large European cohort. J. Psychiatr. Res..

[25] Wittkowski K.M., Dadurian C., Seybold M.P., Kim H.S., Hoshino A., Lyden D. (2018). Complex polymorphisms in endocytosis genes suggest alpha-cyclodextrin as a treatment for breast cancer. PLoS ONE.

[26] Agrahari A.K., Doss G.P.C., Siva R., Magesh R., Zayed H. (2019). Molecular insights of the G2019S substitution in LRRK2 kinase domain associated with Parkinson’s disease: A molecular dynamics simulation approach. J. Theor. Biol..

[27] Agrahari A.K., Priya M.K., Kumar M.P., Tayubi I.A., Siva R., Christopher B.P., Doss C.G.P., Zayed H. (2019). Understanding the structure-function relationship of HPRT1 missense mutations in association with Lesch–Nyhan disease and HPRT1-related gout by in silico mutational analysis. Comput. Biol. Med..

[28] Agrahari A.K., Kumar A., Zayed H. (2018). Substitution impact of highly conserved arginine residue at position 75 in GJB1 gene in association with X-linked Charcot–Marie-tooth disease: A computational study. J. Theor. Biol..

[29] Agrahari A.K., Pieroni E., Gatto G., Kumar A. (2019). The impact of missense mutation in PIGA associated to paroxysmal nocturnal hemoglobinuria and multiple congenital anomalies-hypotonia-seizures syndrome 2: A computational study. Heliyon.

[30] Du H., Brender J.R., Zhang J., Zhang Y. (2015). Protein structure prediction provides comparable performance to crystallographic structures in docking-based virtual screening. Methods.

[31] Rodrigues J.P.G.L.M., Melquiond A.S.J., Karaca E., Trellet M., Van Dijk M., Van Zundert G.C.P., Schmitz C., De Vries S.J., Bordogna A., Bonati L. (2013). Defining the limits of homology modeling in information-driven protein docking. Proteins Struct. Funct. Bioinform..

[32] Sherry S.T., Ward M.-H., Kholodov M., Baker J., Phan L., Smigielski E.M., Sirotkin K. (2001). dbSNP: The NCBI database of genetic variation. Nucleic Acids Res..

[33] Karczewski K.J., Francioli L.C., Tiao G., Cummings B.B., Alföldi J., Wang Q., Collins R.L., Laricchia K.M., Ganna A., Birnbaum D.P. (2019). Variation across 141,456 human exomes and genomes reveals the spectrum of loss-of-function intolerance across human protein-coding genes. bioRxiv.

[34] Choi Y., Chan A.P. (2015). PROVEAN web server: A tool to predict the functional effect of amino acid substitutions and indels. Bioinform.

[35] Adzhubei I.A., Schmidt S., Peshkin L., Ramensky V.E., Gerasimova A., Bork P., Kondrashov A.S., Sunyaev S.R. (2010). A method and server for predicting damaging missense mutations. Nat. Methods.

[36] Kumar P., Henikoff S., Ng P.C. (2009). Predicting the effects of coding non-synonymous variants on protein function using the SIFT algorithm. Nat. Protoc..

[37] Capriotti E., Calabrese R., Casadio R. (2006). Predicting the insurgence of human genetic diseases associated to single point protein mutations with support vector machines and evolutionary information. Bioinform.

[38] Hecht M., Bromberg Y., Rost B. (2015). Better prediction of functional effects for sequence variants. BMC Genom..

[39] Calabrese R., Capriotti E., Fariselli P., Martelli P.L., Casadio R. (2009). Functional annotations improve the predictive score of human disease-related mutations in proteins. Hum. Mutat..

[40] Ashkenazy H., Abadi S., Martz E., Chay O., Mayrose I., Pupko T., Ben-Tal N. (2016). ConSurf 2016: An improved methodology to estimate and visualize evolutionary conservation in macromolecules. Nucleic Acids Res..

[41] Krieger E., Koraimann G., Vriend G. (2002). Increasing the precision of comparative models with YASARA NOVA-a self-parameterizing force field. Proteins Struct. Funct. Bioinform..

[42] Krieger E., Darden T., Nabuurs S.B., Finkelstein A., Vriend G. (2004). Making optimal use of empirical energy functions: Force-field parameterization in crystal space. Proteins Struct. Funct. Bioinform..

[43] Konagurthu A.S., Whisstock J.C., Stuckey P.J., Lesk A.M. (2006). MUSTANG: A multiple structural alignment algorithm. Proteins Struct. Funct. Bioinform..

