# Supplementary material for: Prediction of Functional Consequences of Missense Mutations in ANO4 Gene
Source: Int J Mol Sci. 2021 Mar 8;22(5):2732. doi: 10.3390/ijms22052732 (PMC7962975; doi:10.3390/ijms22052732)
Supplement: Supplementary file 1 [file ijms-22-02732-s001.zip › ijms-1126224-Supplementary material/Supplementary Figures.docx]

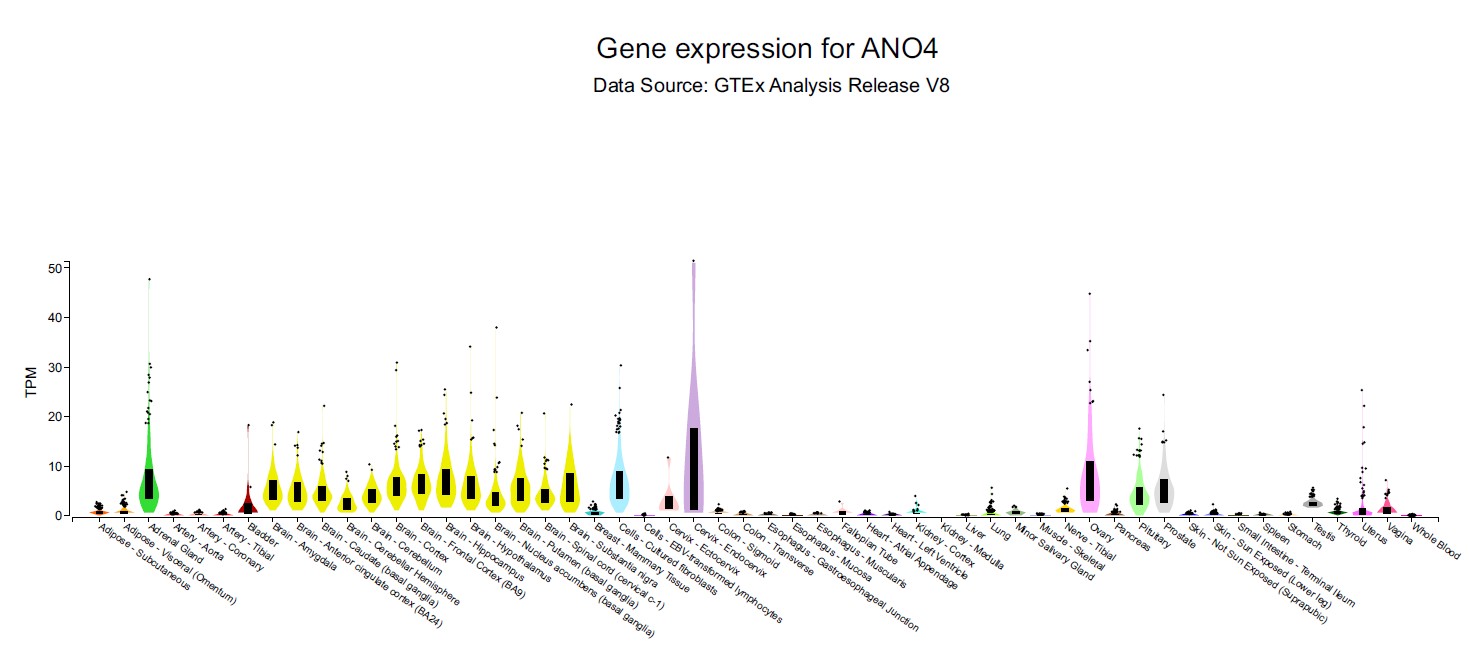


**Figure S1.** Tissue specific expression of human ANO4 in 44 tissues from the Genotype-Tissue Expression (GTEx) project. Expression values are shown in TPM (Transcripts Per Million) calculated from a gene model with isoforms collapsed to a single gene. Box plots are shown as median and 25th and 75th percentiles. Points are displayed as outliers if they are above or below 1.5 times the interquartile range.


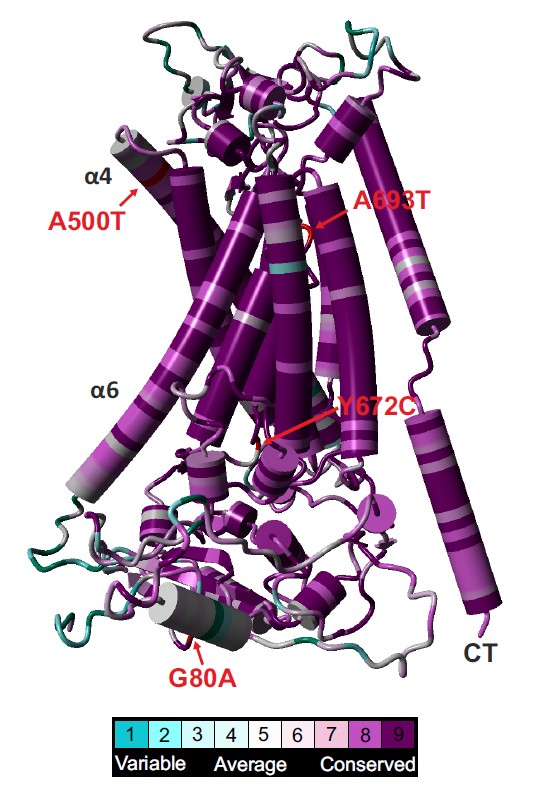


**Figure S2.** Conserved regions of ANO4 detected by ConSurf. The ConSurf algorithm was used to calculate conservation score for the amino acids of human ANO4.
