# Supplementary material for: Prediction of Functional Consequences of Missense Mutations in ANO4 Gene
Source: Int J Mol Sci. 2021 Mar 8;22(5):2732. doi: 10.3390/ijms22052732 (PMC7962975; doi:10.3390/ijms22052732)
Supplement: Supplementary file 1 [file ijms-22-02732-s001.zip › ijms-1126224-Supplementary material/Title of Table S1.docx]

**Table S1.** List of all ANO4 SNPs used in this study
